# Antibody-dependent cellular cytotoxicity-inducing antibodies enhance the natural killer cell anti-cancer response against patient-derived pancreatic cancer organoids

**DOI:** 10.3389/fimmu.2023.1133796

**Published:** 2023-07-14

**Authors:** Nicky A. Beelen, Merel R. Aberle, Virginia Bruno, Steven W. M. Olde Damink, Gerard M. J. Bos, Sander S. Rensen, Lotte Wieten

**Affiliations:** ^1^Department of Internal Medicine, Division of Hematology, Maastricht University Medical Center+, Maastricht, Netherlands; ^2^GROW-School for Oncology and Reproduction, Maastricht University, Maastricht, Netherlands; ^3^Department of Surgery and School of Nutrition and Translational Research in Metabolism (NUTRIM), School of Nutrition and Translational Research in Metabolism, Maastricht University, Maastricht, Netherlands; ^4^Department of Pharmacology and Toxicology, NUTRIM School of Nutrition and Translational Research in Metabolism, Maastricht University, Maastricht, Netherlands; ^5^Department of General, Visceral- and Transplantation Surgery, Rheinisch-Westfälische Technische Hochschule Aachen University, Aachen, Germany; ^6^Department of Transplantation Immunology, Tissue Typing Laboratory, Maastricht University Medical Center+, Maastricht, Netherlands

**Keywords:** natural killer (Nk) cell, pancreatic cancer, organoids, ADCC (antibody dependent cellular cytotoxicity), immunotherapy

## Abstract

**Introduction:**

Pancreatic cancer is associated with poor prognosis, and limited treatment options are available for the majority of patients. Natural killer (NK) cells in combination with antibodies inducing antibody-dependent cell-mediated cytotoxicity (ADCC) could be a highly effective new therapeutic option in pancreatic cancer. Accurate predictive preclinical models are needed to develop successful NK cell immunotherapy. Tumor organoids, in vitro 3D organ-like structures that retain important pathophysiological characteristics of the in vivo tumor, may provide such a model. In the current study, we assessed the cytotoxic potential of adoptive NK cells against human pancreatic cancer organoids. We hypothesized that NK cell anti-tumor responses could be enhanced by including ADCC-triggering antibodies.

**Methods:**

We performed cytotoxicity assays with healthy donor-derived IL-2-activated NK cells and pancreatic cancer organoids from four patients. A 3D cytotoxicity assay using live-cell-imaging was developed and enabled real-time assessment of the response.

**Results:**

We show that NK cells migrate to and target pancreatic cancer organoids, resulting in an increased organoid death, compared to the no NK cell controls (reaching an average fold change from baseline of 2.1±0.8 vs 1.4±0.6). After 24-hours of co-culture, organoid 2D growth increased. Organoids from 2 out of 4 patients were sensitive to NK cells, while organoids from the other two patients were relatively resistant, indicating patient-specific heterogeneity among organoid cultures. The ADCC-inducing antibodies avelumab (anti-PD-L1) and trastuzumab (anti-HER2) increased NK cell-induced organoid cell death (reaching an average fold change from baseline of 3.5±1.0 and 4.5±1.8, respectively). Moreover, combination therapy with avelumab or trastuzumab resulted in complete disintegration of organoids. Finally, inclusion of ADCC-inducing antibodies was able to overcome resistance in NK-organoid combinations with low or no kill.

**Discussion:**

These results support the use of organoids as a relevant and personalized model to study the anti-tumor response of NK cells *in vitro* and the potential of ADCC-inducing antibodies to enhance NK cell effector function.

## Graphical Abstract

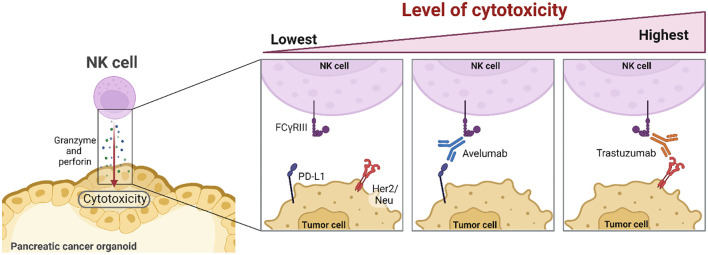

## 1 Introduction

Pancreatic cancer is associated with a poor prognosis and a 5-year survival below 10% (1). Surgery (with adjuvant chemotherapy) is successful in only a minority of patients and therapeutic options have not improved over the past decade. Together with a persistently increasing incidence, pancreatic cancer will likely become the second leading cause of cancer deaths in the US and third in Europe by 2030 (2, 3). Immunotherapy has emerged as a promising treatment option in solid cancer. However, pancreatic cancer is characterized by a dense immunosuppressive stroma, increased inhibitory tumor-associated macrophages, and a low density of endogenous cytotoxic lymphocytes of both the adaptive and innate immune compartments (4). Hence, single agent immune-based treatments are unlikely to be effective in this complex and immunologically ‘cold’ tumor microenvironment (TME) (5, 6).

NK cells are bone-marrow-derived lymphocytes that can recognize and kill tumor cells without having reactivity against healthy cells. Unlike T cells, NK cell activation against target cells does not require prior antigen sensitization (7). However, immune suppression and impaired autologous cytotoxicity have been observed among NK cells in patients with pancreatic cancer, and peripheral endogenous NK cells from pancreatic cancer patients had lower expression of the activating ligands NKG2D and NKp30 (8–11). Moreover, trafficking of NK cells into the TME has been shown to be hampered by lower expression of the chemokine receptor CXCR2 (10). Considering the impaired anti-tumor effector function of patient autologous NK cells, allogenic NK cell therapy may represent a good alternative. However, the infusion of adoptive NK cells alone, while showing minimal toxicities, does not yet achieve sufficient clinical successes in solid cancer types (12–14). NK cell immunotherapy in combination with monoclonal antibodies (mAb) that can induce antibody-dependent cell-mediated cytotoxicity (ADCC) could overcome tumor-mediated immunosuppression and may provide new therapeutic perspectives. ADCC is triggered when the NK cell activating receptor FcγRIIIa (also known as CD16a) binds to the Fc-region of IgG1/IgG3 antibodies, which target surface antigens on potential target cells. Recent data from our group have shown that ADCC can unleash NK cell effector function in a setting that mimics the immunosuppressive TME of breast cancer and multiple myeloma (15, 16). Two well-studied targets for ADCC in solid cancers are human epidermal growth factor receptor 2 (HER2; also known as ErbB-2) and programmed cell death protein ligand (PD-L1; also known as B7-H1 or CD274). The monoclonal antibodies trastuzumab and avelumab, which target HER2 or PD-L1 respectively, are currently FDA approved and have been shown to induce durable tumor remissions in various advanced solid cancers (17, 18). However, immune checkpoint blockade targeting PD-L1 or targeting of HER2 with trastuzumab as monotherapy has been shown to have limited activity in pancreatic cancer (4). The ADCC-inducing capacity and clinical efficacy of these antibodies have not yet been extensively studied in the context of pancreatic cancer. Initial results for the ADCC-inducing capacity of trastuzumab exist, as it was reported that trastuzumab induces ADCC in proportion to HER-2 expression level both *in vitro* and *in vivo* (19–21). To the best of our knowledge, the potential to induce ADCC in pancreatic cancer has not yet been described for avelumab.

Clinical translation of preclinical findings in pancreatic cancer has been impeded because present *in vitro* and *in vivo* scientific models do not accurately reflect the human *in situ* conditions. Long-time passaged 2D cancer cell lines have several drawbacks, such as drifting of pathophysiological features that characterize the original tumor (22, 23). For example, the “classical” versus “basal” subtype differentiation that has important prognostic value in pancreatic cancer is not present in traditional pancreatic cancer cell lines (24). Tumor organoids are *in vitro* 3D organ-like structures that retain important pathophysiological characteristics of the *in-situ* tumor. Compared to 2D cancer cell lines, pancreatic tumor organoids more accurately represent the heterogeneity of tumor epithelial cells and they better recapitulate the complexity of cellular interactions (25, 26).

Scientific models that more accurately represent pancreatic cancer pathophysiology and heterogeneity will aid in optimizing NK cell immunotherapy. The aim of the current study was to assess the cytotoxic potential of allogenic NK cells against human pancreatic cancer organoids. We hypothesized that NK cell anti-tumor responses could be enhanced by including ADCC-triggering antibodies. We demonstrate targeting of pancreatic cancer organoids by NK cells, and that NK cell-mediated cytotoxicity was enhanced by the addition of the ADCC-triggering antibodies trastuzumab and avelumab. Our results support the view that cellular immunotherapies may provide novel treatment options for a largely incurable disease, and hold promise for future patients who do not sufficiently respond to current therapies.

## 2 Methods

### 2.1 2D cell line culture

K562 (ATCC) cells were cultured in IMDM (Gibco, Cat. No. 12440053) supplemented with 10% FCS (TICO Europe, Cat. No. FBSEU500). Capan-2 (ATCC) cells were cultured in RPMI-1640 (Gibco, Cat. No. 61870036) supplemented with 15% FCS (TICO Europe, Cat. No. FBSEU500). MiaPaca-2 (ATCC) cells were cultured in DMEM (Gibco, Cat. No. 31885023) supplemented with 15% FCS (TICO Europe, Cat. No. FBSEU500) and 2.5% horse serum (Gibco, Cat. No. 16050130). All cells were maintained at 37°C in a humidified incubator containing 21% O_2_ and 5% CO_2_ (Sanyo Electric).

### 2.2 3D organoid culture

Previously established (PANCO-11a and PANCO-22a) (27) and newly established (PANCO-45a and PANCO-51) organoid cultures were included in the current study. Tumor tissue from which the new organoids were established, was obtained from patients with pancreatic ductal adenocarcinoma who underwent resection at the Maastricht University Medical Center+ from 2017 to 2021. This study was approved by the local medical ethics committee (METC 2019-0977) and patients provided informed consent prior to inclusion. Organoids were established as previously described (27). Organoids were cultured in three droplets of 15 μL basement membrane extract (BME) (Geltrex^®^ LDEV‐Free Reduced Growth Factor Basement Membrane Matrix, Gibco, Cat. No. 1413202) with 500 μL of organoid Medium 1 or Medium 3 (**Supplementary Table 1**) per well of a 24‐wells non-tissue-treated culture plate (Corning, Cat. No. 351147) and kept in a humidified incubator at 37°C/21% O_2_/5% CO_2_. The medium was changed every 2–3 days and organoids were passaged approximately every 7 days (26–28). Briefly, the BME droplet domes were resuspended in ice‐cold Advanced DMEM/F12 (Gibco, Cat. No. 12634-010) supplemented with Penicillin/streptomycin (50 units/mL penicillin and 50 μg/mL streptomycin, Gibco, Cat. No. 15140-122), 10 mM HEPES (Gibco, Cat. No. 15630-080), and 1x Glutamax (Gibco, Cat. No. 35050-061) (AdvDF+++). The organoids suspensions were pelleted by 5 min centrifugation at 350 x*g* at 4°C and the organoids were mechanically sheared with narrowed glass Pasteur pipettes.

### 2.3 NK cell culture

NK cells were isolated from buffy coats of healthy anonymous volunteers (Sanquin blood bank, Maastricht, The Netherlands). The use of buffy coats does not need ethical approval in the Netherlands under the Dutch Code for Proper Secondary Use of Human Tissue. First, peripheral blood mononuclear cells (PBMCs) were isolated by centrifugation on a Lymphoprep density gradient (Axis-Shield, Cat. No. 07851). From the PBMCs, NK cells were obtained by negative selection, using an NK cell isolation kit (Miltenyi Biotec, Cat. No. 130-092-657) according to the manufacturer’s protocol. The purified NK cells were activated with 1000 U/mL IL-2 (Proleukin, Novartis) and cultured overnight in RPMI-1640 medium (Gibco, Cat. No. 11875093), supplemented with 10% FCS, 100 U/mL penicillin, and 100 µg/mL streptomycin (Gibco, Cat. No. 15140122) in a humidified incubator at 37°C/21% O_2_/5% CO_2_. Effects of organoid growth medium and the culture medium supplement nicotinamide on NK cell cytotoxic capacity were assessed in a 4-hour standard cytotoxicity assay as described before (15). Briefly, the cytotoxic potential of NK cells against K562 was determined by a flow-cytometry based assay. K562 target cells, labeled with CellTracker™ CM-DiI Dye (Thermo Fisher Scientific, Cat. No. C7000), were resuspended in IMDM + 10% FCS or IMDM + 10% FCS + nicotinamide (10 mM). IL-2-activated (1000 U/mL) NK cells were resuspended in RPMI + 10% FCS and co-cultured with K562 cells at a 1:1 or a 5:1 effector-to-target cell (E:T) ratio. After 4-hour co-culture, the cells were stained with Live/Dead^®^ Fixable Aqua Dead Cell Stain Kit (Thermo Fisher Scientific, Cat. No. L34957).

### 2.4 Live cell imaging of NK cell-mediated cytotoxicity

The cytotoxic potential of NK cells against pancreatic tumor organoids was determined using live cell imaging (IncuCyte S3 live cell imaging system (Sartorius)). Three days before the cytotoxicity assay, organoids were dissociated into small organoids using TryplE (Gibco, Cat. No. 12605‐010) supplemented with 10 μM Rho kinase inhibitor and allowed to grow for three days into organoids of homogenous size.

On the day of the cytotoxicity assay, the small organoids were harvested by resuspending the droplet domes in AdvDF+++, and filtered using a 100 µm filter (Greiner Bio-One, Cat. No. 542000). The suspension containing organoids with >100 μm diameter size was discarded. The organoids with ≤100 µm diameter were labeled using 5 µM CellTracker Red CMTPX Dye (Thermo Fisher Scientific, Cat. No. C34552) in AdvDF+++ for 30 min at 37°C. In order to prevent 2D outgrowth of the organoids, 96-wells flat bottom culture plates were coated with ice-cold 100% BME diluted 1:1 in ice-cold PBS (50% BME-PBS scaffold), and incubated at 37°C for at least 30 minutes to solidify before seeding NK cells and organoids. Labeled organoids (≤100 µm diameter) were resuspended in culture medium without Rho Kinase Inhibitor (organoid medium-noROCKi) and counted using a Burker counting chamber. 200 organoids were seeded onto the 50% BME-PBS scaffold and NK cells were immediately added. BME was added to reach a final concentration of 10%. To quantify apoptosis, Caspase-3/7 dye (Sartorius, Cat. No. 4440) was added to each well at a final concentration of 2.5 µM. The NK cells and Caspase-3/7 dye were added immediately after the organoids were seeded and before the 10% BME in the medium was solidified, which would result in inconsistent NK cell migration towards the organoids. Moreover, the co-culture plates were briefly centrifuged (3 min at 300 x*g*) to ensure localization of both organoids and NK cells in one plane. We approximated that a single organoid with a diameter of 100 μm consists of 50 cells. Hence, co-cultures of 2x10^4^ or 5x10^4^ NK cells with 200 organoids represent approximately 2:1 and 5:1 effector-to-target-cell-ratios, respectively.

To assess the effects of ADCC-inducing antibodies, the organoids (≤100 µm diameter) were pre-incubated with ADCC-inducing antibodies or organoid medium-noROCKi in ultra-low attachment round bottom 96 well plates (Corning, Cat. No. 7007) for 30 min at 37°C. Trastuzumab and avelumab were diluted in organoid medium-noROCKi to the following concentrations: 1 µg/mL trastuzumab (Roche), 2 µg/mL avelumab (MedChemExpress, Cat. No. HY-108730).

Organoid-NK cell co-cultures were imaged every 30 min (using the Multi Spheroid scan settings at 10x magnification and one field/well) for 3 days using the IncuCyte S3 live cell imaging system (Sartorius). Organoid segmentation was performed using the IncuCyte S3 Software (Sartorius, version 2019B). A mask selecting the organoids was created based on brightfield images and the fluorescence signal of the CellTrackerRed. The level of apoptosis was quantified from the mean fluorescent intensity of the Caspase-3/7 dye within the organoid masks. As the brightness of the apoptotic signal at baseline varied per well, the level of fluorescence at each time point was divided by the level at baseline in each well. Moreover, the total level of apoptosis over the 24 h cytotoxicity assay was calculated from the area under the curve (AUC) of the caspase-3/7 signal [comparable to (29)]. Images and videos were generated after background subtraction using the IncuCyte S3 software (Sartorius, version 2019B).

### 2.5 Organoid phenotyping with flow cytometry

To determine HER2 and PD-L1 surface expression, organoids were dissociated into single cells using TryplE supplemented with 1:1000 Rho kinase inhibitor. 25,000 single tumor cells were stained with Live/Dead^®^ Fixable Aqua Dead Cell Stain Kit (Thermo Fisher Scientific, Cat. No. L34957) for 30 min on ice. Tumor cells were washed with PBS/EDTA buffer (PBS/2 mM EDTA/0.5% HSA) and stained with mouse anti-human HER2-APC (Neu24.7, BD Biosciences, Cat. No. 340554), anti-PD-L1-PE (29E.2A3, BD, Cat. No. 568079), or matched isotype controls IgG1-APC (MOPC21, BD Biosciences, Cat. No. 550854) and IgG2b,k-PE (27-35, BD, Cat. No. 555058) for 30 min at 4°C. All acquisitions were performed on a BD FACS Canto II. Data were analyzed with FlowJo v10.6.1 (TreeStar).

### 2.6 Determination of KIR – HLA ligand mismatch between NK cell donors and organoid cultures

Genomic DNA was extracted from organoid cultures and NK cells using the QIAamp DNA blood mini kit. The HLA class I genotype of the included organoid cultures and NK cell donors, and the presence of individual KIR genes for the NK cell donors were determined by Luminex- sequence-specific oligonucleotides (SSO) (One Lambda). The NK cell donor genotypes and licensing statuses are presented in **Supplementary Table 2**. HLA-Bw4 is recognized by KIR3DL1, HLA-C1 by KIR2DL2/3, and HLA-C2 by KIR2DL1. The matched and mismatched NK cell populations were identified based on the genotypic expression of the respective HLA epitope in organoid cultures. Moreover, mismatches were only taken into account when NK cells could be licensed through the mismatched KIR, i.e. the corresponding HLA gene was also genotypically present in the NK cell donor. Matched and mismatched conditions between the NK cell and organoid cultures are presented in **Supplementary Table 3**.

### 2.7 Statistical analyses

Statistical analyses were performed with GraphPad Prism 9 software using Kruskal-Wallis test or paired ANOVA with Tukey’s multiple comparisons test when appropriate (described per analysis). Values are expressed as mean ± standard error or deviation, where applicable. P-values of <0.05 were considered statistically significant. Group size, population information, and error bar definitions are indicated in the figure legends.

## 3 Results

### 3.1 Activated NK cells can migrate towards organoids and are not hampered in their cytotoxic function by organoid culture medium

To investigate NK cell cytotoxicity towards pancreatic cancer organoids, we established a three-dimensional co-culture method that can be monitored in real-time using brightfield and fluorescent imaging. The included organoid cultures demonstrated heterogeneous phenotypes: one grew in solid cell clusters (PANCO-45a), while others (PANCO-11a, PANCO-22a, PANCO-51) had the more common cystic structures (27). To test the capability of NK cells to migrate towards organoids within culture medium containing BME, we first optimized the seeding procedure of the organoids to be used in the cytotoxicity assays. Based on a previous publication (30), we seeded organoid suspensions on top of a solidified BME scaffold. Immediately after seeding (t=0 h), the organoids, showed a normal morphology (**Supplementary Figure 1A**). Moreover, we observed that this setup was sufficient to prevent organoid 2D outgrowth for the duration of the cytotoxicity assays (24 hours, **Supplementary Figure 1A**).

**Figure 1:**
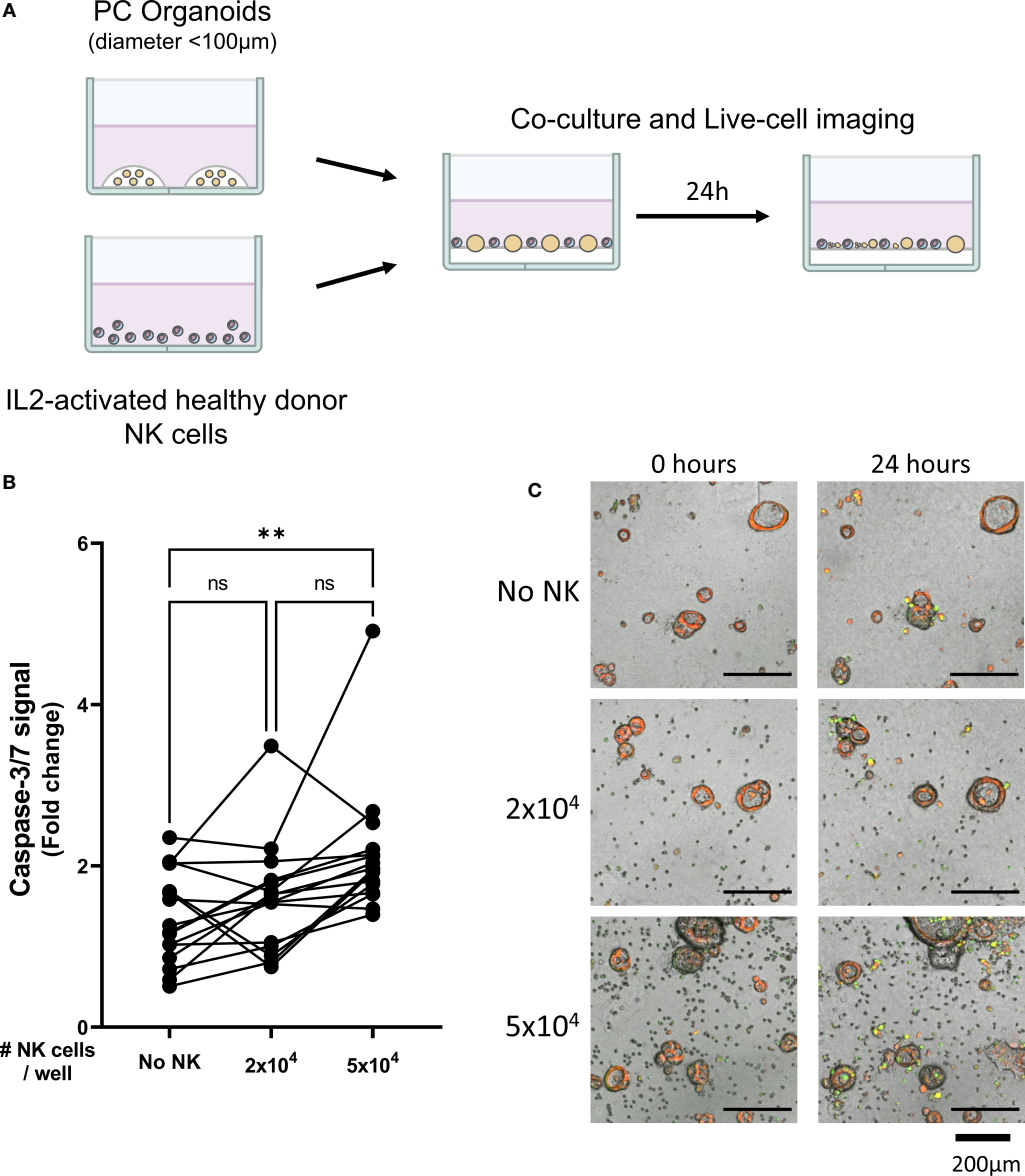
Healthy donor-derived NK cells can target pancreatic cancer organoids. **(A)** Pancreatic organoid cultures are enzymatically and mechanically sheared, and monitored for 3 days until cytotoxicity assay. NK cells are isolated from healthy donors and activated with 1000 U/mL IL2. NK cell cytotoxicity assays and live-cell imaging is performed for 24 h. **(B)** Cytotoxicity assays were performed with 2x10^4^ or 5x10^4^ NK cells and 200 organoids per well; apoptotic signal of the organoids at t=24 h is shown as fold change calculated as the signal at t=24 h divided by signal at t=0 (h) Each dot represents the average of duplicates from one NK cell – organoid culture combination. N=16; 5 NK donors & 4 organoid cultures pooled. Value of 1 indicates no increased apoptosis compared to baseline t=0 (h) **P < 0.01, ns = not significant by paired ANOVA. **(C)** Representative images of co-culture with NK cells and pancreatic cancer organoids after 0, 12, and 24 h with increasing NK cell numbers. Organoids are labeled red. Caspase-3/7 apoptotic signal in green.

Next, we optimized the seeding procedure of the NK cells to be used in the cytotoxicity assays. Initially, the organoids were seeded and allowed to settle for 24 h, during which the 10% BME in the organoid medium solidified as well. After 24 h, the NK cells in suspension were added and apoptosis was quantified by live cell imaging (**Supplementary Figure 1B**). When the BME within the organoid medium was pre-solidified, NK cells migrated towards the organoids but localized at different depths within the culture well. This hampered focusing on the correct plane during live cell imaging. Subsequently, we seeded NK cells and organoids at the same time on top of the BME scaffold. This resulted in the localization of both NK cells and organoids in one plane (**Supplementary Figure 1C**), allowing accurate focusing during live cell imaging. When NK cells were tracked for the duration of the cytotoxicity assay, efficient trafficking of NK cells towards organoids could be observed. We therefore continued with this setup (**Figure 1A**).

Organoids grow in medium containing nicotinamide, which has been shown to inhibit the cytotoxic capacity of the NK92 cell line (30). We therefore tested whether NK cell viability and cytotoxicity were hampered by the nicotinamide in the organoid medium. NK cells were co-cultured with 2D grown K562 cells, a cell line that is highly sensitive to NK cells and that is commonly used as positive control cell line for NK cell killing. Flowcytometric analysis showed that the addition of nicotinamide to the K562 culture medium did not affect NK cell viability nor did it significantly hamper killing of K562 during a standard cytotoxicity assay (**Supplementary Figures 1D, E**).

### 3.2 NK cell lytic activity induces pancreatic tumor organoid apoptosis, which increases with increasing NK cell doses

To assess the cytotoxic potential of NK cells in the 3D co-culture system, we performed cytotoxicity assays with four independent pancreatic tumor organoid cultures and different doses of NK cells. After 24-hours of co-culture, organoid 2D outgrowth increased (**Supplementary Figure 1A**). Hence, the cytotoxicity assay was analyzed up to 24 hours as the later time points yielded less accurate data due to 2D growth.

Organoid death was quantified by assessing the level of apoptosis in organoids cultured alone (**Video 1**) or together with 2x10^4^ or 5x10^4^ NK cells (**Videos 2**, **3**). The addition of 2x10^4^ NK cells to 200 organoids did not significantly increase the level of apoptosis in the organoids (1.6 ± 0.7 vs 1.4 ± 0.6, p=0.860 vs No NK cell control (**Figures 1B, C**). However, co-culturing 5x10^4^ NK cells with 200 organoids significantly increased cytotoxicity among the organoids compared to organoids not co-cultured with NK cells (2.1 ± 0.8 vs 1.4 ± 0.6, p=0.005) (**Figures 1B, C**). Overall, NK cells induced more organoid apoptosis at higher NK-to-organoid ratios, demonstrating that NK cells can effectively target patient-derived pancreatic cancer organoids.

### 3.3 Patient-derived pancreatic tumor organoids express variable levels of PD-L1 and HER2

**Figure 2:**
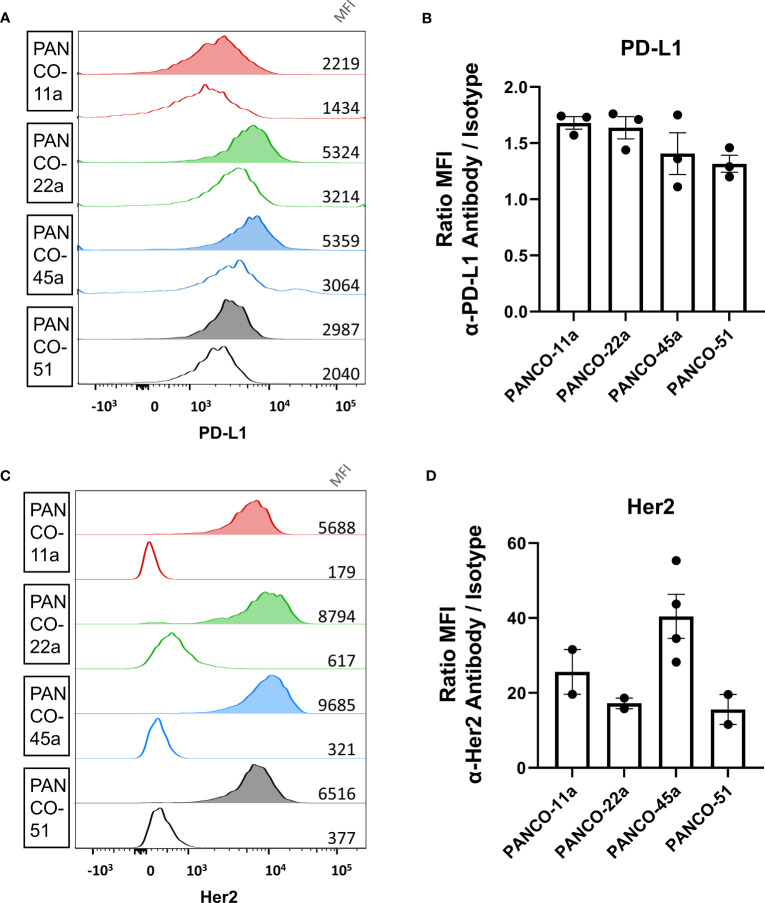
Validation of PD-L1 and HER2 expression on the surface of organoid cells. **(A, C)** Representative histograms of **(A)** PD-L1 and **(C)** HER2 staining of pancreatic tumor organoid cells. Clear histograms show isotype negative control. Median fluorescent intensity is depicted. **(B, D)** Ratio of mean fluorescent intensity (MFI) of signal from the **(B)** α-PD-L1 antibody or **(D)** α-HER2 antibody divided by the MFI of the respective isotype controls ± SD. Individual dots represent replicate experiments.

NK cell induced apoptosis of organoids did not result in complete eradication of organoids and surviving organoids were able to continue expanding (**Supplementary Figure 2**). Therefore, we aimed to enhance NK cell-induced cytotoxicity by ADCC using the clinically available mAbs against PD-L1 (avelumab) and HER2 (trastuzumab). We first validated the surface expression of PD-L1 and HER2 protein on patient-derived pancreatic tumor organoid cultures by flow cytometry (gating strategy in **Supplementary Figure 3A**). All organoid cultures displayed PD-L1 surface expression (**Figures 2A, B**). The ratio between the median fluorescence intensity (MFI) from the α-PD-L1 antibody divided by the MFI of the isotype controls indicated that organoid cultures PANCO-11a and PANCO-22a had slightly higher PD-L1 expression compared to PANCO-45a and PANCO-51 (1.7 ± 0.1 and 1.6 ± 0.2 vs 1.4 ± 0.3 and 1.3 ± 0.1), respectively). Furthermore, all organoid cultures tested expressed HER2 on the cell surface (**Figures 2C, D**). PANCO-45a consistently had the highest HER2 expression (40.5 ± 11.8), followed by PANCO-11a (25.6 ± 8.4). PANCO-22a and PANCO-51 had the lowest expression (17.2 ± 2.0 and 15.6 ± 5.7) (**Figures 2C, D**). We compared the PD-L1 and HER2 expression levels of the organoids with two conventional pancreatic cancer cell lines: Capan-2 and MiaPaca-2. PD-L1 expression was comparable between the organoid cultures and MiaPaca-2, while Capan-2 had approximately 10-fold higher expression compared to the organoid cultures and the MiaPaca-2 cell line (**Supplementary Figure 3B**). Comparing HER2 expression on organoids with cell lines indicated that PANCO-45a had the highest expression level. Capan-2 cells had comparable expression levels with PANCO-11a, whereas MiaPaca-2 had the lowest expression levels, which was even lower compared to the organoid cultures (**Supplementary Figure 3C**). As all organoids expressed PD-L1 and HER2 on their surface, treatment with ADCC-inducing antibodies may increase NK cell cytotoxicity.

### 3.4 ADCC-inducing antibodies enhance NK cell lytic activity against pancreatic organoids coated with trastuzumab or avelumab

We next assessed whether the lytic activity of donor NK cells could be enhanced *in vitro* following pre-treatment of organoids with the ADCC-inducing antibodies avelumab (anti-PD-L1) or trastuzumab (anti-HER2). In the absence of NK cells, pre-incubation of organoids with trastuzumab or avelumab did not increase apoptosis, indicating the absence of direct toxicity mediated by the monoclonal antibodies (**Supplementary Figures 4A, B**).

**Figure 3:**
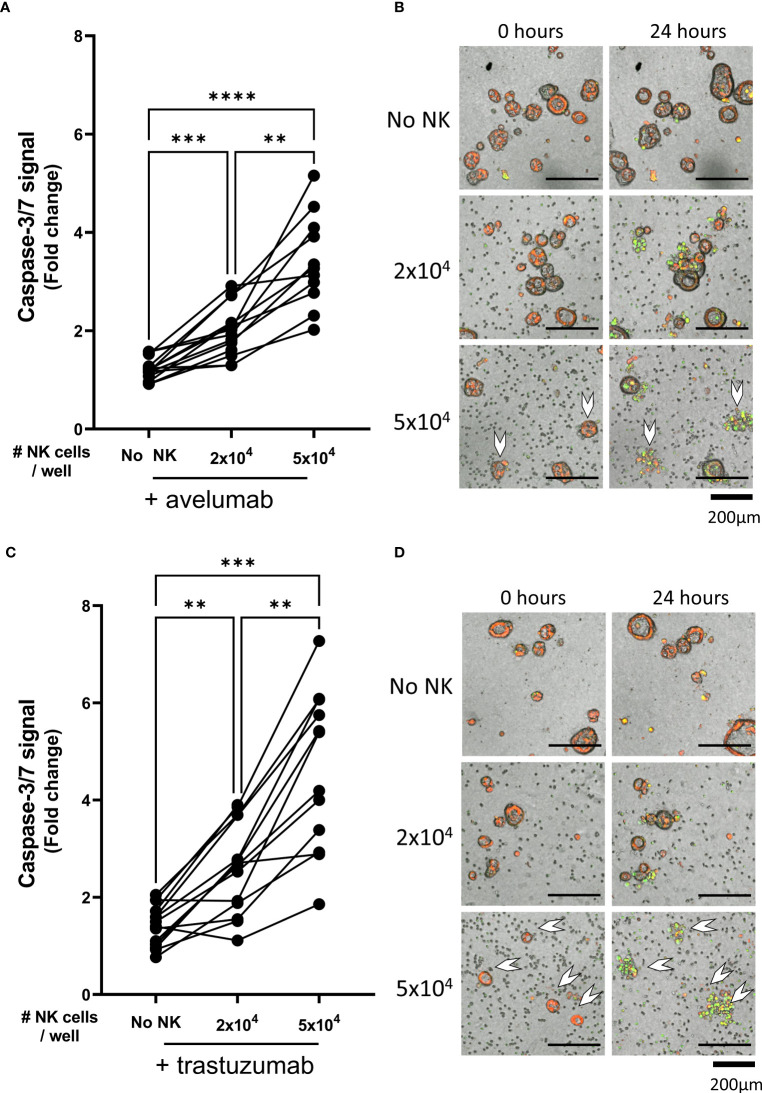
NK cell efficacy against organoids pre-treated with trastuzumab or avelumab. Cytotoxicity assays with. **(A)** avelumab or **(C)** trastuzumab were performed with 2x10^4^ or 5x10^4^ NK cells and 200 organoids per well; the apoptotic signal of the organoids at t=24 h is shown as fold change calculated as the signal at t=24 h divided by signal at t=0 h. Each dot represents the average of duplicates from one NK cell – organoid culture combination. N=16; 5 NK donors & 4 organoid cultures pooled. Value of 1 indicates no increased apoptosis compared to baseline t=0 h. **P < 0.01, ***P <0.001, ****P <0.0001 by paired ANOVA. **(B, D)** Representative images of co-cultured NK cells and pancreatic cancer organoids after 0 and 24 h. Organoids are labeled red. Caspase-3/7 apoptotic signal in green. Completely disintegrated organoids indicated with white arrows.

As compared to the condition with only avelumab, co-culture with 2x10^4^ NK cells after pre-incubation with avelumab significantly increased organoid apoptosis (1.2 ± 0.2 vs 2.0 ± 0.5, p=0.001) (**Figures 3A, B**). Moreover, the level of apoptosis was dependent on the E:T ratio, increasing up to 5.2-times after co-culture with 5x10^4^ NK cells (3.4 ± 0.9, p<0.0001 vs avelumab only, p=0.003 vs 2x10^4^ NK cells + avelumab). Moreover, combination therapy with 5x10^4^ NK cells and avelumab resulted in complete disintegration of some organoids (**Figure 3B** white arrows).

Compared to the trastuzumab only control, organoid apoptosis was significantly increased upon pre-incubation with trastuzumab in the presence of 2x10^4^ NK cells (1.4 ± 0.4 vs 2.5 ± 0.9, p=0.005) (**Figures 3C, D**). The level of apoptosis was dependent on the NK cell number since addition of 5x10^4^ NK cells resulted in up to 7.3-times higher apoptosis compared to baseline (4.6 ± 1.6), which was significantly higher compared to the level of apoptosis observed with trastuzumab only or 2x10^4^ NK cells with trastuzumab (p=0.0001 vs trastuzumab only, p=0.001 vs 2x10^4^ NK cells + trastuzumab). Moreover, combination therapy with 5x10^4^ NK cells and trastuzumab resulted in complete disintegration of the majority of organoids (**Figure 3D**).

### 3.5 Trastuzumab induces stronger NK cell lytic activity against pancreatic organoids compared to avelumab

**Figure 4:**
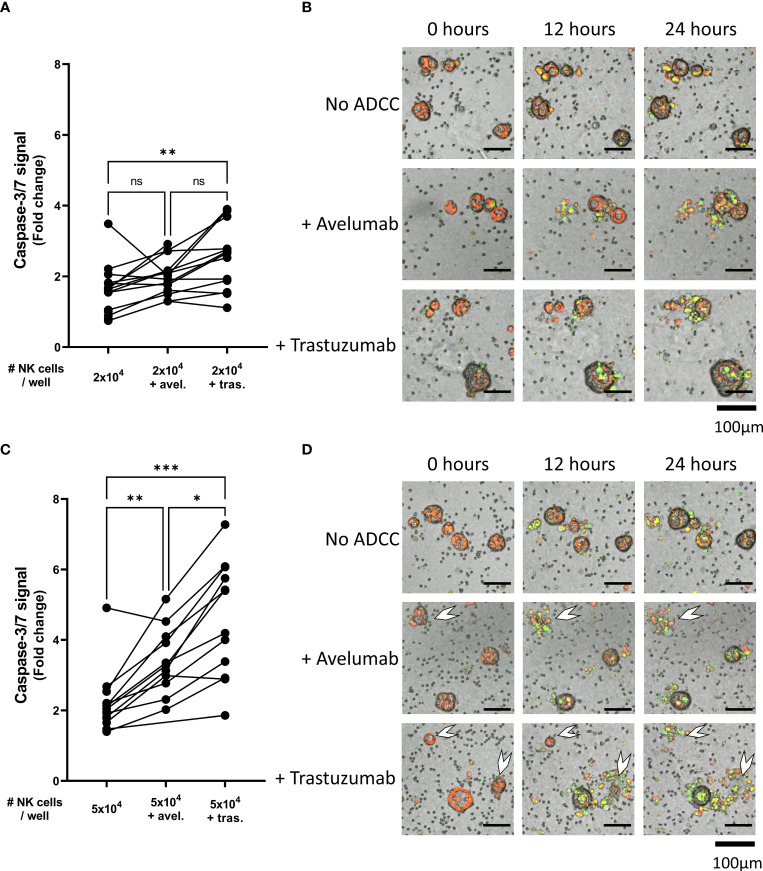
NK cell cytotoxic efficacy against organoids with or without ADCC. Cytotoxicity assays were performed with **(A, B)** 2x10^4^ or **(C, D)** 5x10^4^ NK cells against 200 organoids per well and apoptotic signal of the organoids at t=24 h are shown as fold change calculated as the signal at t=24 h divided by signal at t=0 h. Each dot represents the average of duplicates from one NK cell – organoid line combination. N=16; 4 NK donors & 4 organoid lines pooled. Value of 1 indicates no increased apoptosis compared to baseline t=0 h. *P < 0.05, **P < 0.01, ***P < 0.001 by paired ANOVA. **(B, D)** Representative images of co-culture with NK cells and pancreatic cancer organoids after 0, 12, and 24 h. Organoids are labeled red. Caspase-3/7 signal in green. Completely disintegrated organoids indicated with white arrows. ns, not significant.

Comparing the ADCC conditions with the conditions without ADCC-inducing antibodies showed that the level of NK cell-mediated cytotoxicity was significantly enhanced upon pre-incubation with either avelumab or trastuzumab. On average, the level of organoid apoptosis was low when 2x10^4^ NK cells were used (**Figures 4A, B**). Moreover, pre-incubation of the organoids with avelumab did not significantly increase apoptosis compared to the condition without ADCC (2.0 ± 0.5 vs 1.6 ± 0.7, p=0.4). However, the inclusion of trastuzumab increased apoptosis compared to controls without ADCC (2.5 ± 0.9 vs 1.6 ± 0.7, p=0.005), but not compared to the avelumab condition (2.5 ± 0.9 vs 2.0 ± 0.5, p=0.16). Co-culture with 5x10^4^ NK cells in combination with either avelumab or trastuzumab resulted in higher levels of apoptosis compared to the conditions without ADCC-inducing antibodies (2.1 ± 0.8 for No ADCC vs 3.4 ± 0.9, p=0.004 and vs 4.6 ± 1.6, p=0.001 for avelumab and trastuzumab, respectively) (**Figures 4C, D**). Moreover, the level of apoptosis induced by trastuzumab was higher compared to that induced by avelumab (4.6 ± 1.6 vs 3.4 ± 0.9, p=0.01) (**Figures 4C, D**). In addition to increasing the caspase-3/7-derived fluorescent signal, combination therapy with NK cells and ADCC-inducing antibodies resulted in complete disintegration of organoids, which was especially clear for the conditions with trastuzumab (**Figures 3B–D**, **4D** white arrows, Video 3-6). While variances in cytotoxic capacity amongst the NK cell donors were present, we did not observe NK cell donors with consistently higher cytotoxic capacity (**Supplementary Figure 5A**). Instead, color coding of organoid cultures suggested the presence of NK sensitive and resistant organoids (**Supplementary Figure 5B**).

### 3.6 Live cell imaging identifies NK-cell combination therapy with trastuzumab as most effective

**Figure 5:**
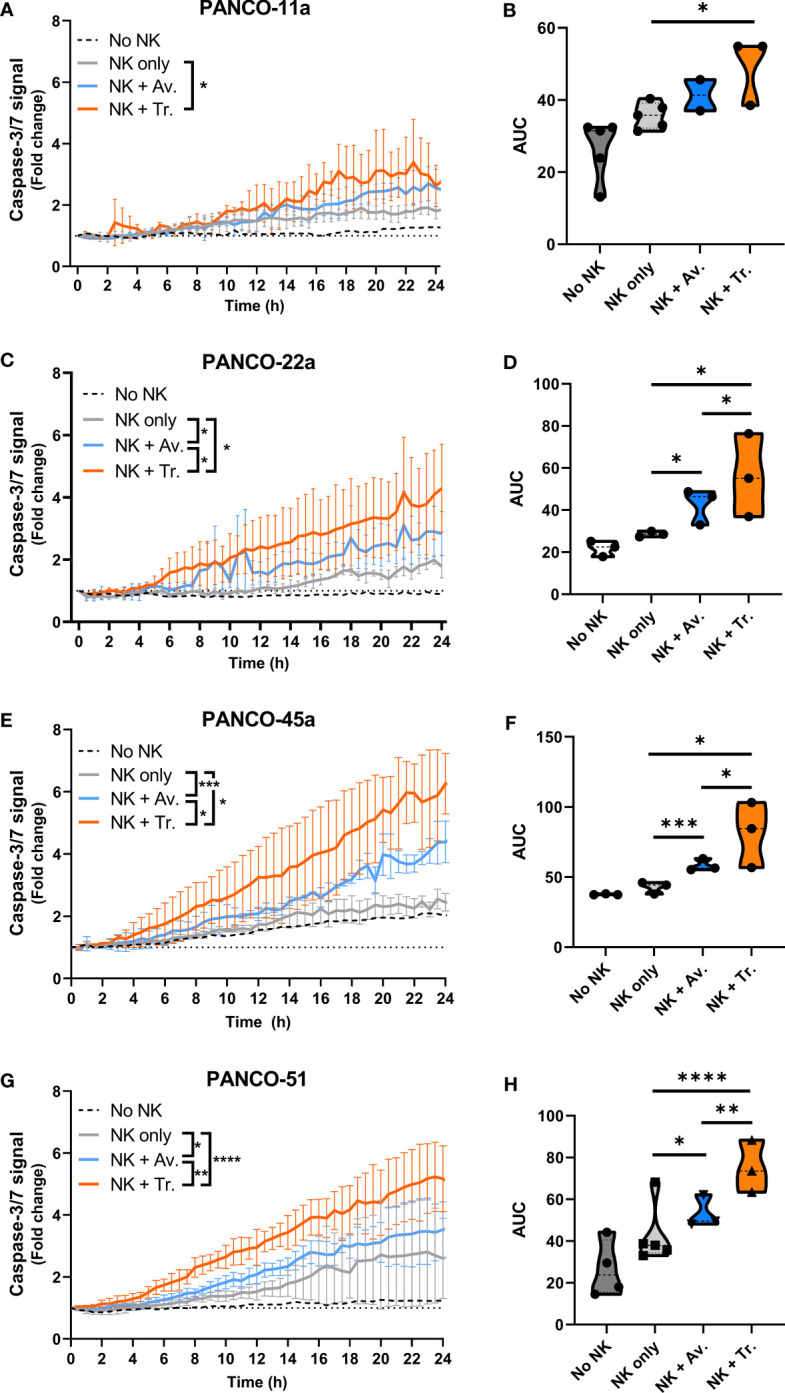
NK cell cytotoxic efficacy with or without ADCC against the different organoid cultures. **(A, C, E, G)** Cytotoxicity assays were performed with 5x10^4^ NK cells and 200 organoids per well, and apoptotic signal ( ± SD) of the organoids over 24 h is shown as fold change calculated as the signal at t=24 h divided by signal at t=0 h. No NK cell value indicates data from all organoids pooled. Value of 1 indicates no increased apoptosis compared to baseline t=0 h. Results from all NK cell donors pooled. N=3-5 NK donors. *P < 0.05, **P < 0.01, ***P <0.001, ****P <0.0001 by comparing area under the curve (AUC) by ANOVA. **(B, D, F, H)** AUC quantification.

We next studied the efficacy of NK cell therapy in combination with ADCC-inducing antibodies by tracking the level of organoid apoptosis during the 24 h live cell imaging assay. For this, the total level of apoptosis for each organoid culture that was induced by 5x10^4^ NK cells with or without ADCC was calculated as the area under the curve (AUC) in the 24 h cytotoxicity assay. In organoid cultures PANCO-11a, PANCO-22a, PANCO-45a, and PANCO-51, the combination of NK cells with trastuzumab resulted in significantly higher total apoptotic signal compared to the No ADCC control (p=0.01, p=0.02, p=0.01, and p<0.0001, respectively) (**Figure 5**). For organoid culture PANCO-11a, avelumab increased the total level of apoptosis, but this increase did not reach significance (p=0.06). The total level of apoptosis induced by NK cells with trastuzumab was higher compared to avelumab, but this again did not reach the level of significance (p=0.0524) (**Figures 5A, B**). For organoid cultures PANCO-22a, PANCO-45a, and PANCO-51, the total apoptotic signal from avelumab-induced ADCC was significantly higher compared to the conditions without ADCC (p=0.02, p=0.0003, and p=0.015, respectively) (**Figures 5C–H**). Moreover, for these organoid cultures, total apoptosis mediated by trastuzumab-induced ADCC was significantly higher compared to the avelumab-induced ADCC (p=0.04, p=0.0295, and p=0.001, respectively) (**Figures 5C–H**).

### 3.7 Organoids from different pancreatic cancer donors show heterogeneous sensitivity towards NK cell-mediated cytotoxicity

**Figure 6:**
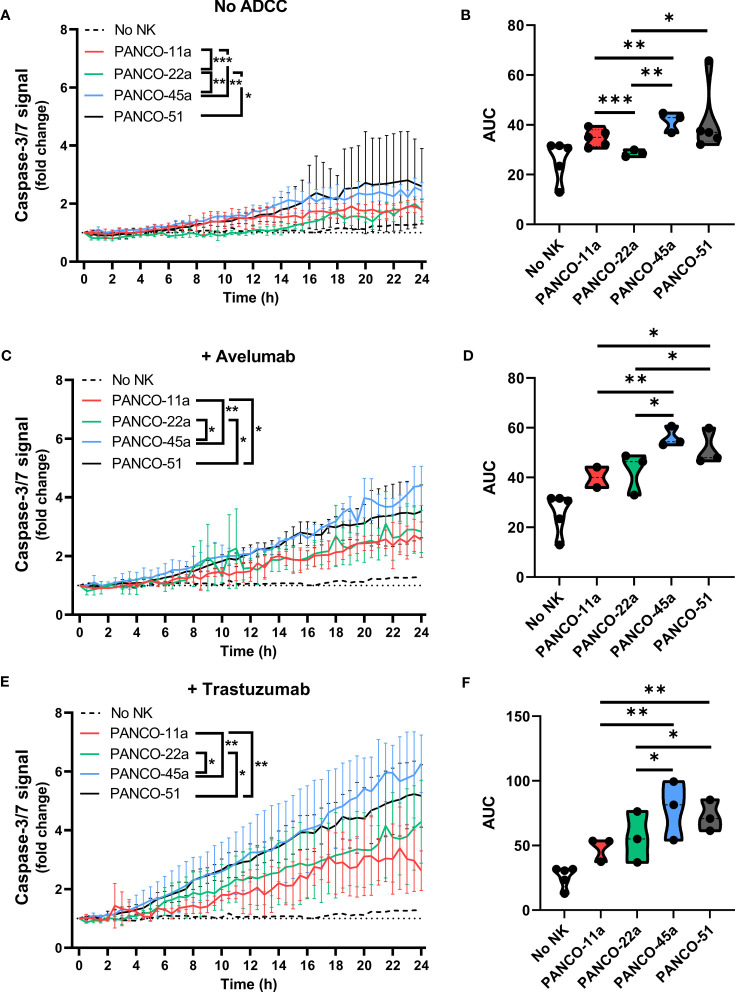
Heterogeneous susceptibility of the different organoid cultures to NK cells and ADCC combination therapy. **(A, C, E)** Cytotoxicity assays were performed **(A)** without ADCC-inducing antibodies, **(C)** with avelumab or **(E)** with trastuzumab and 5x10^4^ NK cells plus 200 organoids per well. Apoptotic signal ( ± SD) of the organoids over 24 h is shown as fold change calculated as the signal at t=24 h divided by signal at t=0 h. No NK cell value indicates data from all organoids pooled. Value of 1 indicates no increased apoptosis compared to baseline t=0 h. Results from all NK cell donors pooled. N=3-5 NK donors. *P < 0.05, **P < 0.01, ***P <0.001 by comparing area under the curve (AUC) by ANOVA. **(B, D, F)** AUC quantification.

Next, we addressed potential heterogeneity in response to NK cell treatment between the pancreatic cancer organoids. Treatment of organoids with only NK cells resulted in significant differences in total apoptotic signal between PANCO-11a and PANCO-22a (34.8 ± 0.6 vs 28.6 ± 0.4, p=0.001); PANCO-11a and PANCO-45a (34.8 ± 0.6 vs 41.4 ± 0.6, p=0.003); PANCO-22a and PANCO-45a (28.6 ± 0.4 vs 41.4 ± 0.6, p=0.001); and PANCO-22a vs PANCO-51 (28.6 ± 0.4 vs 41.4 ± 2.1, p=0.02) (**Figures 6A, B**).

The addition of avelumab to NK cell treatment resulted in higher total apoptotic signal for PANCO-45a and PANCO-51 compared to PANCO-11a and PANCO-22a (56.1 ± 0.8 and 51.5 ± 1.1 vs 40.1 ± 0.8 and 42.7 ± 1.4, p=0.003 and p=0.01; and p=0.01 and p=0.03, respectively). Therefore, PANCO-45a and PANCO-51 were the organoid cultures with the highest response to NK cell with avelumab treatment from the four organoid cultures tested (**Figures 6C, D**). Interestingly, the level of PD-L1 expression was not in line with the level of sensitivity of the organoids towards NK cell treatment with avelumab. PANCO-45a and PANCO-51 had the lowest PD-L1 expression levels, while these organoid cultures were most responsive to NK cell therapy with avelumab.

The heterogeneity in response between the pancreatic cancer organoid cultures was further emphasized in the conditions treated with NK cells and trastuzumab. The addition of trastuzumab to NK cell treatment resulted in higher total apoptotic signal for PANCO-45a and PANCO-51 compared to PANCO-11a and PANCO-22a (78.4 ± 2.6 and 72.6 ± 1.6 vs 48.1 ± 1.7 and 56.1 ± 2.4, p= 0.001 and p=0.003; and p=0.01 and p=0.04, respectively). Similar to the observations with avelumab, PANCO-45a and PANCO-51 were the organoid cultures with the highest response to NK cell with trastuzumab treatment from the four organoid cultures tested (**Figures 6E, F**). However, the level of HER2 surface expression was not in line with the level of response after addition of trastuzumab, as the level of HER2 expression was higher for PANCO-11a compared to PANCO-51, whereas the apoptotic signal upon NK cells with trastuzumab treatment was consistently higher for PANCO-51 compared to PANCO-11a.

### 3.8 Impact of the number of mismatches between killer cell immunoglobulin receptors and human leukocyte antigen ligands on the level of sensitivity towards NK cell-mediated cytotoxicity

**Figure 7:**
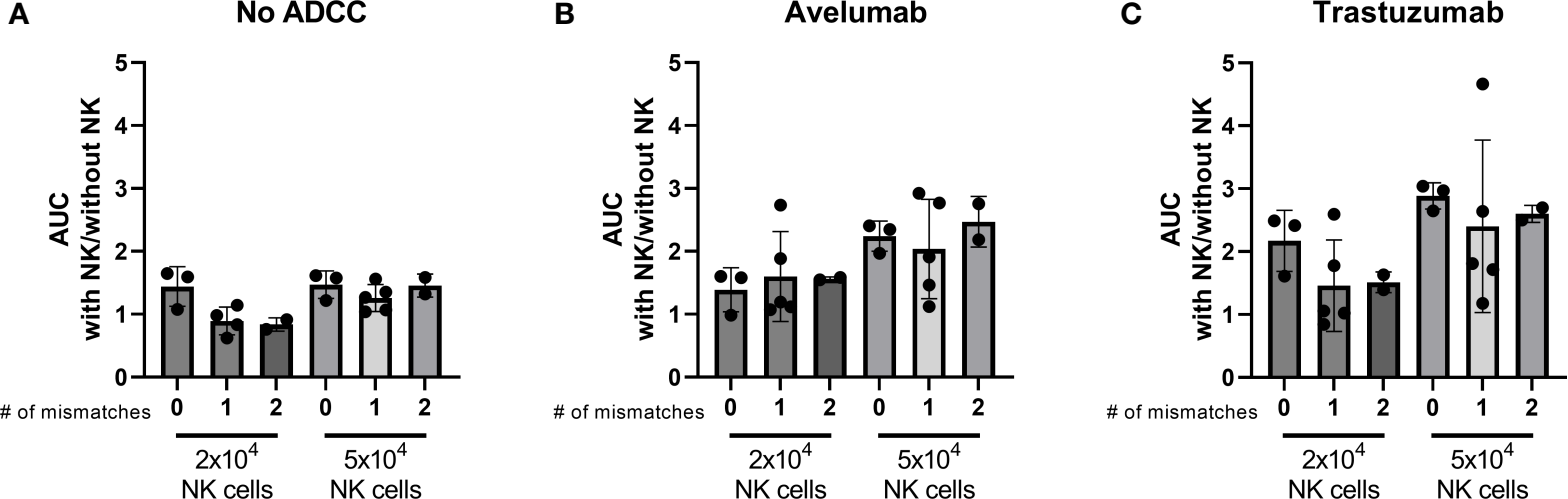
Effect of the number of HLA-KIR mismatches on the level of apoptosis induced by licensed NK cells after 24 h NK-organoid co-culture. A mismatch was defined as a KIR for which the corresponding HLA ligand was present in the donor while it was absent in the organoid. A match was defined as a KIR for which the corresponding HLA ligand was present in both the donor and in the organoid. Level of cytotoxicity induced by **(A)** NK cells alone, **(B)** NK cells combined with avelumab, or **(C)** with trastuzumab is presented as the area under the curve for the cytotoxicity assay with 2x10^4^ or 5x10^4^ NK cells divided by the area under the curve for the cytotoxicity assay without NK cells. Dots represent individual combinations of organoid cultures with NK cell donors, which are grouped based on the number of licensed mismatches at the genetic level. N=10; 3 NK cell donors against 4 organoid cultures, missing value for NK cell donor 1 condition with 2x10^4^ NK cells and without ADCC.

NK cell activation is determined by the balance between inhibitory and activating signaling it receives from a potential target cell. The interaction between the human killer cell immunoglobulin-like receptors (KIR) and human leukocyte antigen (HLA)-Class I is an important inhibitory signal for NK cells and thus an important regulator of NK cell activity. Introducing a mismatched situation between the NK donor-KIR and patient-HLA would create a situation where inhibitory signaling is removed, thereby reducing the amount of necessary activating signaling. We hypothesized that an increased number of KIR-HLA ligand mismatches between NK donors and organoids could result in stronger NK cell effector function. Therefore, we performed HLA and KIR typing as an initial approach to decipher a potential mechanism that could affect organoid sensitivity to NK cell treatment. The combinations of organoid cultures and NK cell donors were ranked based on the number of mismatches (ranging from no mismatches to two mismatches). We assessed the ability of 2x10^4^ and 5x10^4^ NK cells to induce apoptosis, calculated as area under the curve (AUC), and compared it to the AUC of the No NK cell control. We did not observe a clear relationship between the number of matched HLA-KIR pairs versus mismatched pairs at the genomic level and the level of NK cell cytotoxicity (**Figure 7**).

## 4 Discussion

In this work, we describe the potential of NK cells to target patient-derived primary pancreatic cancer organoids in a 3D setting and show that the NK cell cytotoxic potential against pancreatic cancer organoids can be enhanced by using clinically available monoclonal antibodies with the ability to trigger ADCC by targeting of HER2 and PD-L1. The existence of NK cell-sensitive and -resistant organoid cultures observed in the present study, is beneficial for better understanding of the heterogeneous response to NK cell immunotherapy and for studies aiming to improve the response to NK cells. Previous studies demonstrated the conservation of patient-specific gene expression profiles between organoids and the original tumor tissue (31, 32). If characteristics mediating NK cell response are also conserved, the organoid cultures may be predictive for patient response to NK cell immunotherapy.

We observed that the limited cytotoxicity of adoptive NK cells against pancreatic cancer organoids could be enhanced by including the ADCC-inducing antibodies avelumab and trastuzumab, targeting PD-L1 and HER2, respectively. We did not observe direct toxicities for the tumor cells upon incubating the organoids with either avelumab or trastuzumab alone. Although several studies on checkpoint inhibition through the PD-L1/PD-1-axis or monotherapies with trastuzumab in the pancreatic cancer setting are ongoing, these approaches have so far failed to elicit efficacy (33, 34), which is in line with our current *in vitro* data. While ADCC has been identified as an important pathway to enhance NK cell anti-tumor function for a long time, limited data is available in the context of pancreatic cancer. In one study, combination therapy with PBMCs or NK cells and trastuzumab has been shown to induce ADCC-mediated activity towards pancreatic cancer cell lines, which was proportional to their level of HER2 expression (20) and supports our data in showing the potential for combination regimens that utilize the power of NK cell-mediated ADCC.

Blockade of the immunosuppressive PD-L1/PD-1 axis is another mechanism which can enhance anti-tumor effects, especially in T cells (35). However, unlike T cells, NK cells in the peripheral blood of healthy individuals do not express PD-1 on their surface, with the exception of a fraction of cytomegalovirus positive individuals (36). Hence, we do not expect that the observed enhanced response with avelumab in our study was mediated through immune checkpoint blockade on NK cells, but rather exclusively through induction of ADCC. Similarly, we do not expect NK cell activation through binding of avelumab onto PD-L1 expressed on NK cells (37), as we did not observe PD-L1 expression on NK cells in our setting (unpublished data). The potential for NK cell therapy in combination with PD-L1 targeting has not yet been studied in the setting of pancreatic cancer, which, according to our observations, could be a new immunotherapeutic approach for pancreatic cancer patients. Using an ADCC-inducing antibody variant to block PD-L1-PD1 interaction, like avelumab, for that purpose could have a dual effect by triggering NK cell-mediated ADCC and by lower inhibitory signaling in PD1 positive T cells.

In our study, we observed heterogeneity in the response of organoids to NK cells. Moreover, we did not observe an association between the level of ADCC and the level of HER2 or PD-L1 expression. In a first attempt to understand the underlying mechanism, we evaluated KIR-HLA matching status as the interaction between inhibitory HLA ligands and NK cells is an important inhibitory checkpoint for NK cells. However, heterogeneity between organoids could not be explained by the number of genotypic KIR-HLA ligand mismatches between NK cell donors and the organoid cultures. In this experimental setup, we were not able to assess the KIR-HLA match versus mismatch effect at the level of KIR surface expression, neither did we determine the response in NK cells that exclusively expressed matched KIRs vs mismatched KIRs by, for example, using NK cell clones or looking at single cell degranulation status (15). There are two reasons why this would be important in order to fully capture the relevance of matching status. First, KIR null alleles exist, hence the genotypic presence of a receptor does not always result in protein expression (38). Second, the percentage of single KIR expressing NK cells is relatively low and NKG2A, the inhibitory receptor for HLA-E expressed on 50-80% of NK cells, may dominate the response in bulk analysis (38). In addition to KIR and NKG2A, NK cell activation is controlled by a large array of inhibitory- and activation receptors. Hence, other receptor-ligand pairs could also influence NK cell and ADCC efficacy. Future effort will focus on deciphering the observed heterogeneous response of patient derived organoids to NK cells using broader and unbiased efforts, such as RNA sequencing or functional CRISPR/Cas9 screens (39). Such additional studies could provide rationale to test additional strategies to interfere with dominant inhibitory receptor-ligands such as KIR-HLA class I and NKG2A-HLA-E interaction (15, 40) and novel combinations of monoclonal antibodies to improve clinical efficacy of NK cells.

In the current study, we established a co-culture system in which NK cell-induced apoptosis within tumor organoids can be quantified in real-time and where single cell digestion of the organoids is not required. So far, limited options are available for real-time analysis of NK cell cytotoxicity using organoids in their 3D composition, and these have not been combined with monoclonal antibodies to induce ADCC. The majority of the preclinical work studying NK cell immunotherapy in solid cancer has been performed in 2D monolayer cancer cell lines or spheroids. For instance, Gopal and colleagues (19) assessed the efficacy of NK cell combination with trastuzumab in spheroids generated from a pancreatic cancer cell line. Compared to the cytotoxicity assays performed with 2D monolayer cultures, the enhanced cytotoxicity upon trastuzumab was virtually absent when the same cells were cultured as 3D spheroids (19). Spheroids consist of homotypic cell types that do not fully represent *in vivo* cellular interactions and polarization, which are better preserved in organoids. Moreover, as organoid formation is primarily driven by internal developmental processes that support long-term expansion of the structures, the genetic features of the original tumor are better retained (26). While organoid models have been frequently used to test and predict sensitivity versus resistance to anti-cancer drugs, only a limited number of studies evaluated their potential as model systems to test immunotherapeutic approaches (30, 41). Our data show that it is feasible to use organoids for testing the effect of cellular immunotherapies in combination with other therapeutic agents.

One major advantage of organoids, as compared to model systems involving primary tumor cells cultured in monolayer, is that organoids better represent the tissue of origin. The basement membrane extract used in organoid culture allows for physiological cell-matrix protein interactions that mimic the cellular environment *in vivo*, something that is not feasible within standard stiff plastic culture dishes (42). Hence, our data showing the potential of NK-antibody combination therapy in primary organoids are highly encouraging for the further development of NK cell-based approaches in pancreatic cancer. However, even though organoid models better represent cell-cell interactions, it does not comprise the full complexity of the *in vivo* tumor and current efforts in the field are focused on generating models that include more factors from the *in vivo* environment. For instance, organoid models lack the influence of other cell types present in the tumor microenvironment, such as cancer-associated fibroblasts (CAFs). CAFs are considered to be a significant driver of tumor development, and affect response to treatment and (NK cell) immunosuppression (43, 44). Our pancreatic cancer organoid cultures consist out of pancreas ductal epithelial cells and there was no visual indication that stromal cells were present in the cultures that were used for our experiments. During establishment of organoid cultures, stromal cells that are present in the dissociated tumor tissue suspension attach to the bottom of the culture plate and are excluded from subsequent culture, as they are not harvested by, for example, trypsin (26, 27). Therefore, further *in vitro* reconstruction of the TME by including CAFs into the existing organoid culture systems may be of significant relevance. Current technical advances, such as chip-platforms or bioprinting, could allow for combining multiple relevant immunosuppressive hurdles and improve the concept of a complete *in vitro* TME even further. Novel technologies such as organoids or chip-platforms could also address the ability of NK cells to migrate towards and infiltrate into tumors (45), which is still a challenge for NK cell therapy for solid cancers.

In the current study, NK cell induced apoptosis was assessed by the level of active caspase-3/7. NK cells mainly mediate their anti-tumor effector function through induction of tumor cell apoptosis, which involves Caspase-3/7 (46, 47). In future experiments, additional readouts could be included such as measurement of organoid shape and recruitment and accumulation of NK cells in proximity to the organoids. Adaptation of the protocol can allow for further experimentation where, for example, organoids can be harvested and digested to identify the level of infiltration by NK cells (48). Another challenge for organoid modelling of *in vivo* tumors is that it remains unclear to what extend organoids can recapitulate the (genetic) heterogeneity observed intratumorally. Whether specific organoid culture conditions may support growth of particular subpopulations of tumor cells remains to be investigated. In parallel to organoid models, the use of murine models remains critical to further explore the recruitment towards and effectivity against tumor cells in an *in vivo* setting. However, each pancreatic cancer preclinical mouse model will come with drawbacks as no model will perfectly recapitulate the human immune system.

Our ultimate goal is to develop an immunotherapy using healthy donor-derived NK cells to support the autologous immune system. This NK cell product is to be administered as an “off-the-shelf” system, eliminating the need for individual processing of each patient’s autologous NK cells. This significantly shortens the time until the start of treatment and allows for the modification of the NK cell product to optimize their *in vivo* efficacy. It has been described that endogenous tumor-infiltrating NK cells are inactive and lose FcγRIIIa expression, which limits the potential of ADCC (9, 10) [Reviewed in: (49)]. This could be an underlying reason why monotherapy using ADCC-inducing drugs, but without the addition of allogeneic NK cells, has not shown significant clinical efficacy (4, 50, 51). Whether adoptive NK cells undergo the same fate is yet to be determined, but approaches such as genetic modifications of NK cells may support their infiltration and prevent their inactivation (10).

To conclude, we observed that healthy donor NK cells effectively target organoids derived from pancreatic cancer patients when combined with ADCC-inducing antibodies. This represents a novel treatment approach in pancreatic cancer, which currently lacks effective treatment options. Moreover, our platform may be used to screen pancreatic cancer patients and predict their response to NK cell therapy.

## Data availability statement

The original contributions presented in the study are included in the article/**Supplementary Material**. Further inquiries can be directed to the corresponding author.

## Author contributions

Conceptualization: NB, SR, and LW. Methodology: NB and MA. Investigation: NB, MA, VB. Analysis: NB and MA. Recourses: SR, SOD, GB, and LW. Writing- original draft preparation: NB. Writing- review and editing: MA, SOD, GB, SR, and LW. All authors have read and agreed to the published version of the manuscript. All authors contributed to the article and approved the submitted version.
