# Supplementary material for: Antibody-dependent cellular cytotoxicity-inducing antibodies enhance the natural killer cell anti-cancer response against patient-derived pancreatic cancer organoids
Source: Front Immunol. 2023 Jul 14;14:1133796. doi: 10.3389/fimmu.2023.1133796 (PMC10375290; doi:10.3389/fimmu.2023.1133796)
Supplement: Supplementary file 1 [file DataSheet_1.pdf]

Supplementary Material

Antibody-dependent cellular cytotoxicity-inducing antibodies enhance the natural killer cell anti-cancer response against patient-derived pancreatic cancer organoids

Nicky A. Beelen, Merel R. Aberle, Virginia Bruno, Steven W.M. Olde Damink, Gerard M.J. Bos, Sander S. Rensen[A], Lotte Wieten[A,*]

*** Correspondence:** Lotte Wieten: l.wieten@mumc.nl

1 Supplementary Data

Supplementary Material should be uploaded separately on submission. Please include any supplementary data, figures and/or tables.

Supplementary material is not typeset so please ensure that all information is clearly presented, the appropriate caption is included in the file and not in the manuscript, and that the style conforms to the rest of the article.

2 Supplementary Figures and Tables

For more information on Supplementary Material and for details on the different file types accepted, please see here.

2.1 Supplementary Figures

Video 1-3: Organoids cultured alone (Video 1) or together with $2x10^4$ or $5x10^4$ NK cells (Video 2 +3). Videos created by combining images taken 30 min apart over 24 h. Organoids are labeled red with CellTrackerRed. Caspase-3/7 apoptotic signal in green.

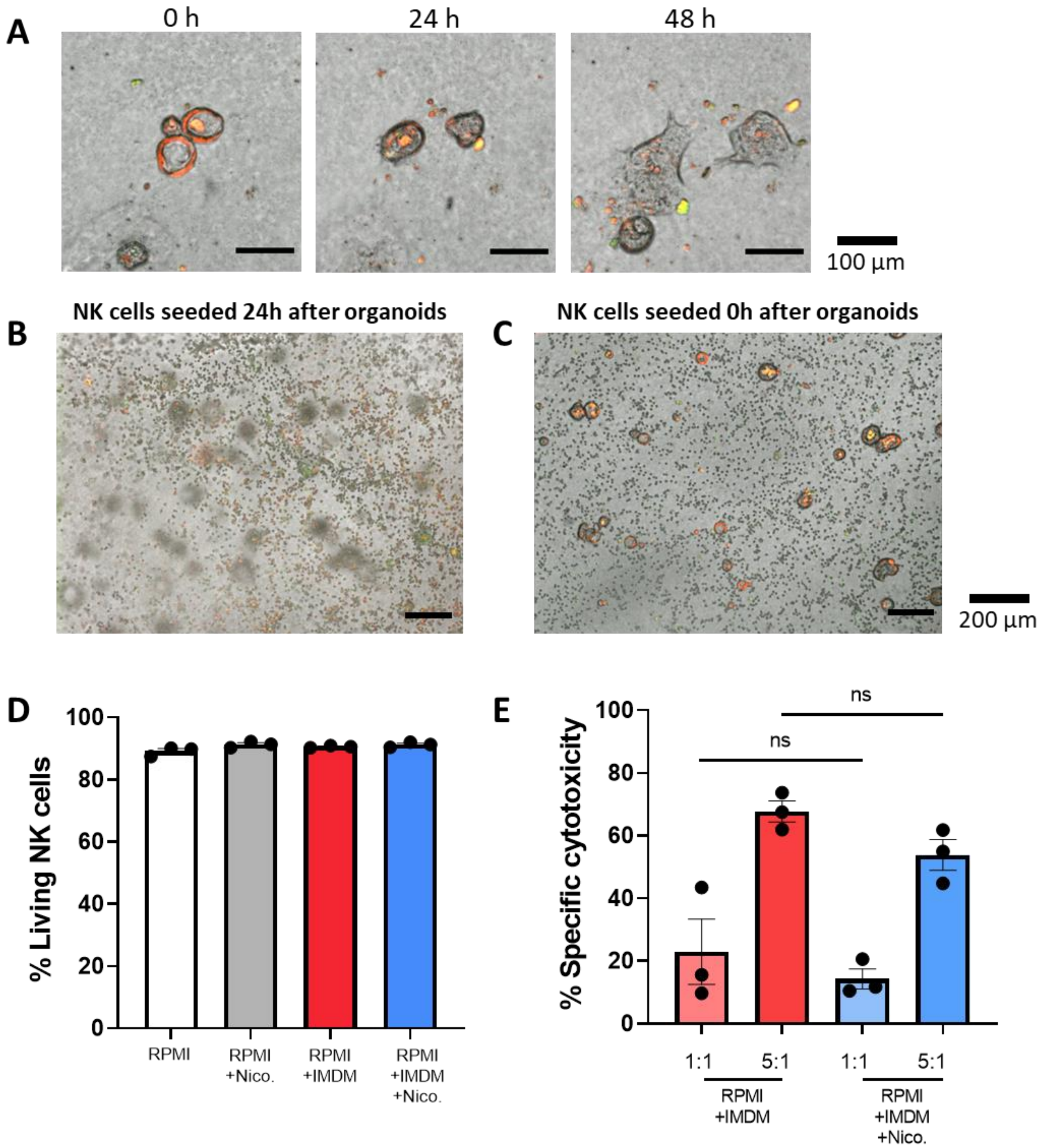

Supplementary Figure 1: Establishing natural killer cell cytotoxicity assays against pancreatic tumor organoids. (A) Seeded organoids on top of 100% BME diluted 1:1 with PBS. Organoids are labeled red with CellTrackerRed. Caspase-3/7 apoptotic signal in green. Images taken at t = 0 h, 24 h, and 48 h. **(B-C)** Co-culture of 200 pancreatic cancer organoids and $5x10^4$ natural killer cells. **(B)** Organoids are seeded 24h before the NK cells, whereas **(C)** organoids and NK cells are seeded simultaneously. Organoids are labeled red with CellTrackerRed. Caspase-3/7 apoptotic signal in green. Images taken at t = 1 h after NK cell seeding. **(D)** Effect of organoid culture medium components on viability of NK cells. NK cells were cultured in indicated media for 4h. Values derived from three

replicate NK cell donors. **(E)** Effect of organoid culture medium component nicotinamide on NK cell cytotoxic capacity against K562 cells. Nico = nicotinamide; GCU = Green Calibrated Units.

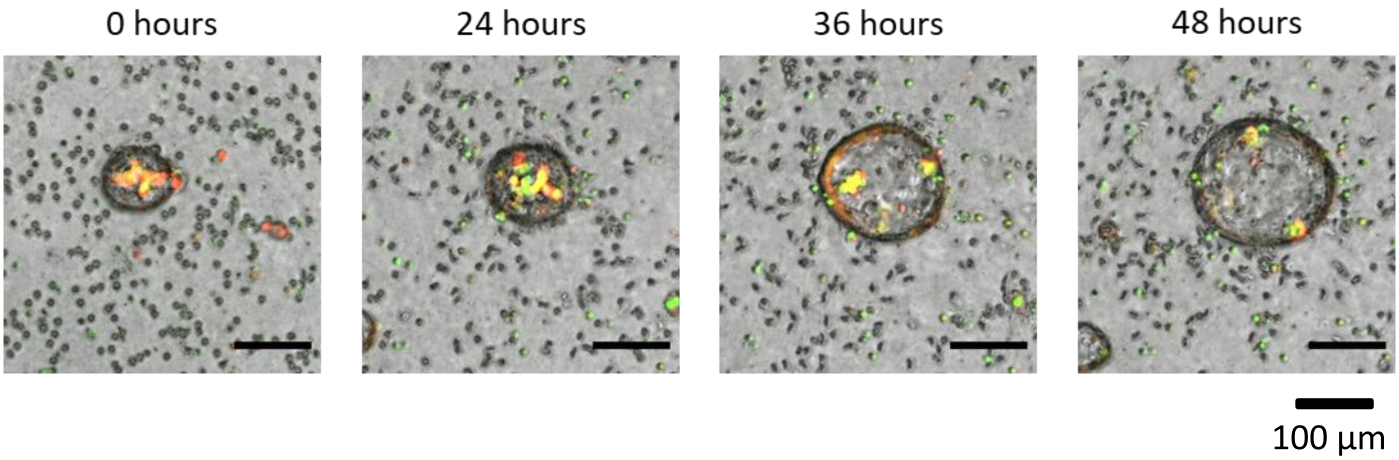

Supplementary Figure 2: NK cells induce apoptosis in organoids but do not completely eradicate organoids. Expansion of organoids during NK cell treatment. Organoids are labeled red with CellTrackerRed. Caspase-3/7 apoptotic signal in green.

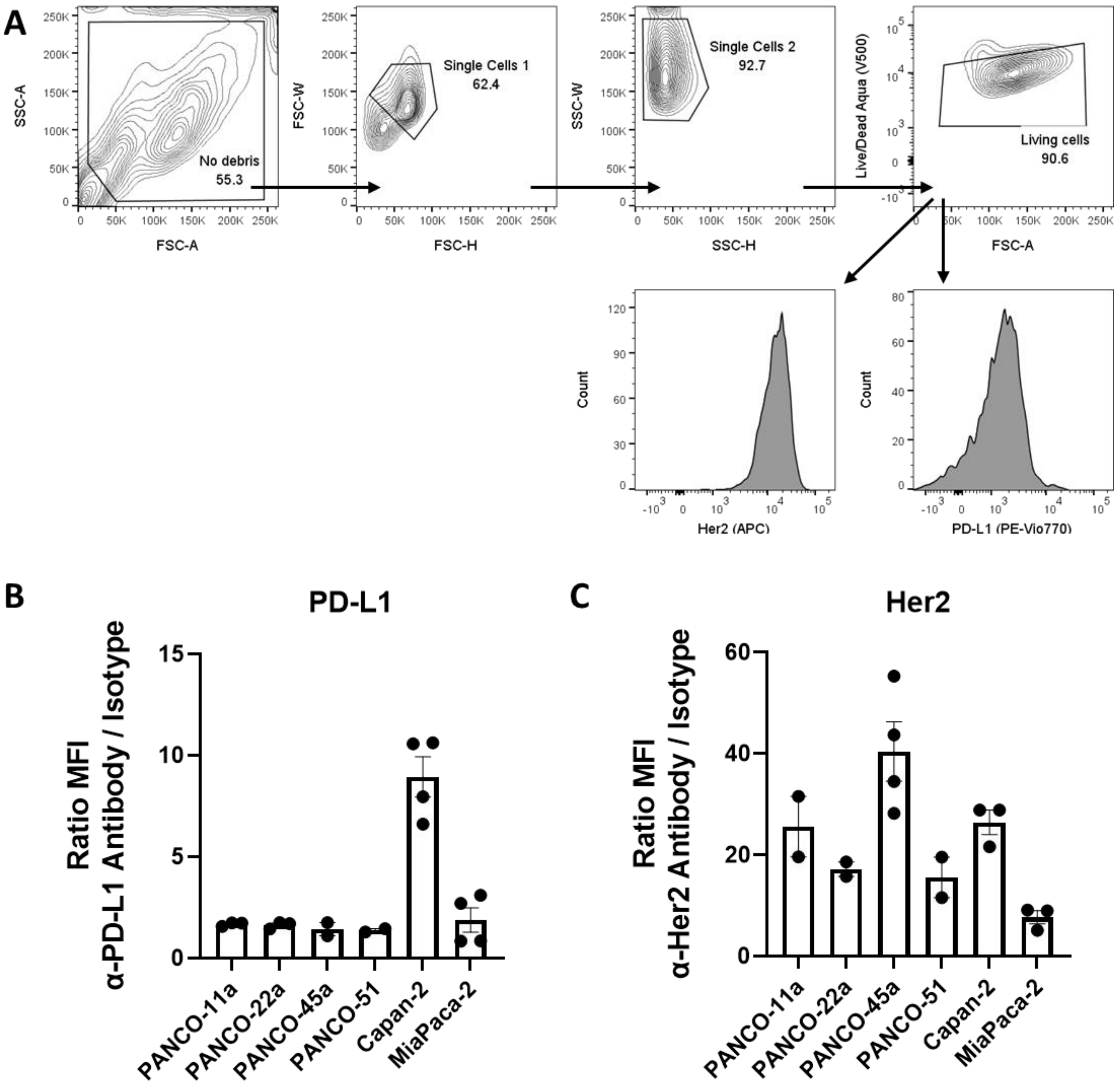

Supplementary Figure 3: Gating strategy for organoid phenotyping and comparison to pancreas cancer cell lines. (A) From left to right: debris, duplets, and dead cells are gated out. Next, cells positive for either PD-L1 or Her2 are identified based on negative controls (isotype). **(B+C)** Comparison of **(B)** α-PD-L1 and **(C)** HER2 expression on organoids with expression levels of two pancreas cancer cell lines. Ratio of mean fluorescent intensity (MFI) of signal from the **(B)** α-PD-L1 antibody or **(C)** α-HER2 antibody divided by the MFI of the respective isotype controls ± SD is depicted. Individual dots represent replicate experiments.

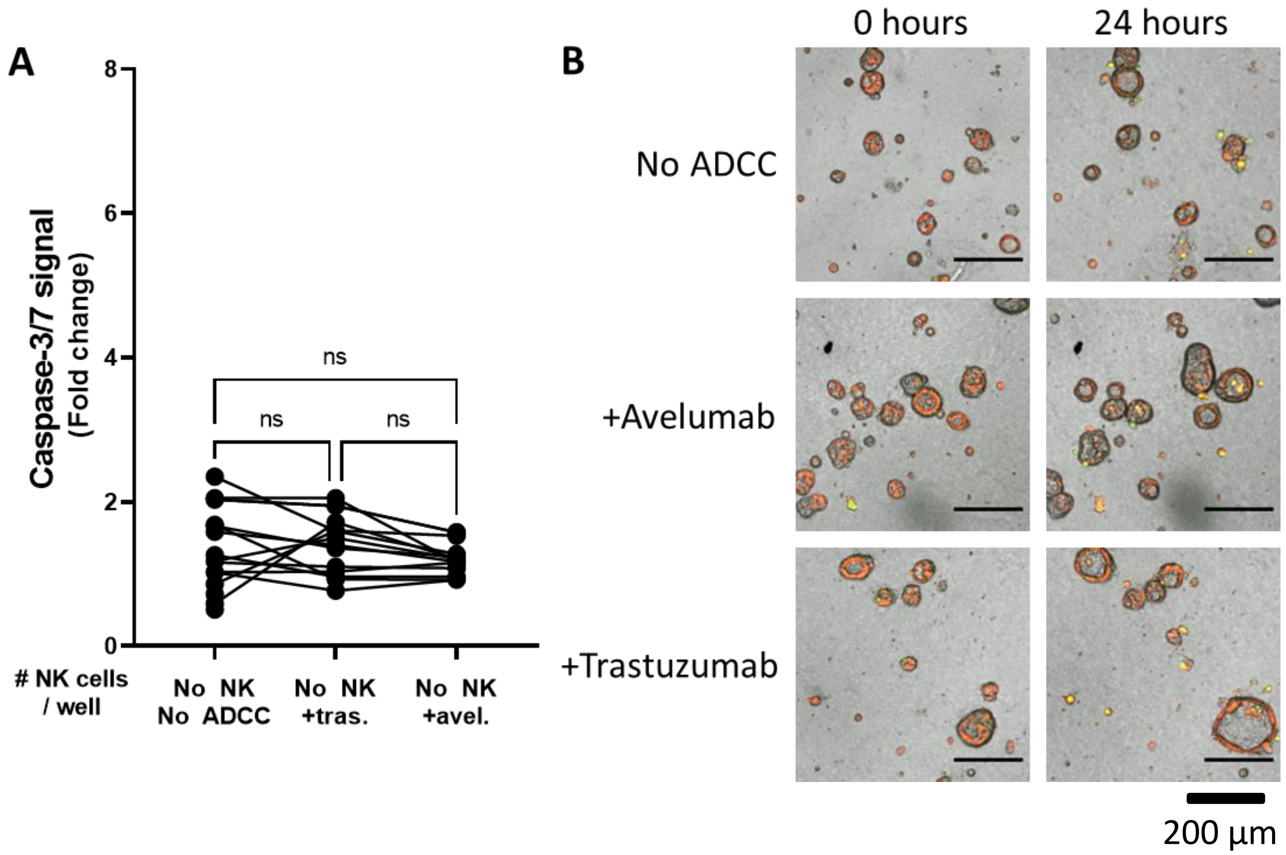

Supplementary Figure 4: No spontaneous cytotoxicity by avelumab or trastuzumab. **(A)** Evaluation of direct cytotoxicity of trastuzumab and avelumab. Fold-change of Caspase-3/7 signal at t=24h compared to t=0h. N= 16; 5 NK donors & organoid cultures pooled. Value of 1 indicates no increased apoptosis compared to baseline. No significant differences by paired ANOVA. **(B)** Representative images of co-culture with NK cells and pancreatic cancer organoids after 0 and 24 h. Organoids are labeled red. Caspase-3/7 signal in green.

Video 4-6: Organoids cultured with NK cells only (Video 4), or with NK cells in combination with and avelumab (Video 5) or trastuzumab (Video 6). Videos created by combining images taken 30 min apart over 24 h. Organoids are labeled red with CellTrackerRed. Caspase-3/7 apoptotic signal in green.

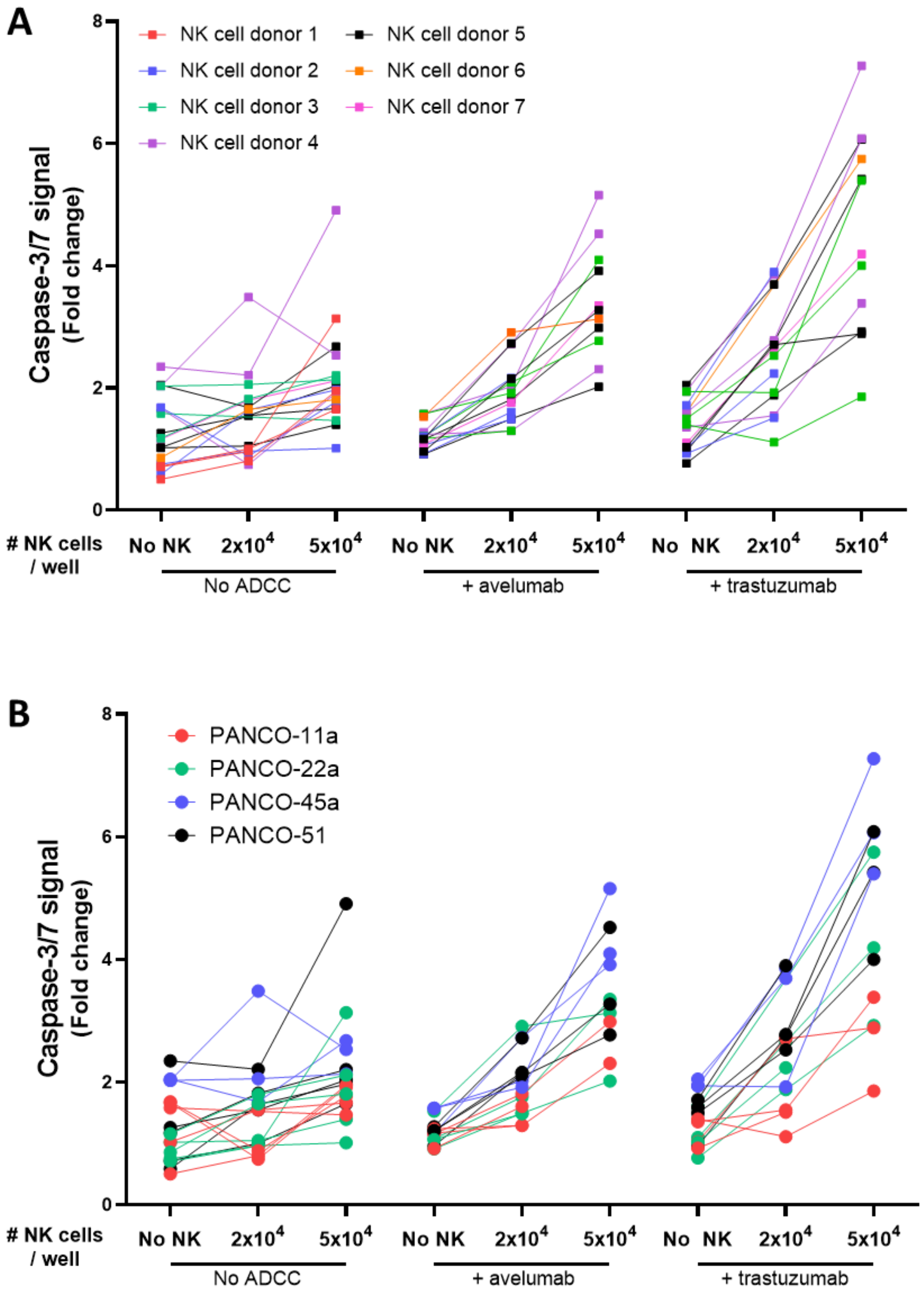

Supplementary Figure 5: NK cell efficacy against organoids pre-treated with trastuzumab or avelumab color coded by (A) different NK cell donors or (B) different organoid cultures. Cytotoxicity assays against 200 organoids per well were performed without ADCC-inducing antibodies, with avelumab or with trastuzumab, and without or with $2x10^4$ or $5x10^4$ NK cells; the apoptotic signal of the organoids at t = 24 h is shown as fold change from t = 0 h (signal at t=24h

divided by signal at t=0h). Each dot represents the average of duplicates from one NK cell – organoid culture combination. N=16; 3-5 NK donors & 4 organoid cultures pooled. Value of 1 indicates no increased apoptosis compared to baseline t=0 h. **(A)** All cytotoxicity assays performed with the same NK donor have the same color. **(B)** All cultures from the same patient-derived organoid have the same color.

2.2 Supplementary Tables

Supplementary Table 1: Organoid growth medium composition

Components	Cat. No.	Tumor 1 medium	Tumor 3 medium
Adv. DMEM/F12 +++	Gibco®, #12634-010	75% (v/v)	25% (v/v)
Noggin CM	Conditioned medium*	10% (v/v)	10% (v/v)
Rspo1 CM	Conditioned medium*	10% (v/v)	10% (v/v)
Wnt3a CM	Conditioned medium*	-	50% (v/v)
B27 supplement	Gibco®, #17504-044	1:50	1:50
EGF Recombinant Mouse Protein	Gibco®, #PMG8043	5 ng/ml	5 ng/ml
N-acetyl cysteine	Sigma-Aldrich, #A9165	1.25 mM	1.25 mM
Primocin	InvivoGen, #ant-pm-1	1:500	1:500
Nicotinamide	Sigma-Aldrich, #N0636	10 mM	10 mM
A83.01	Sigma-Aldrich, #SML0788	-	500 nM
FGF10	Sino-Biological, #10573-HNAE	100 ng/ml	100 ng/ml
Gastrin	Sigma-Aldrich, #G9145	10 nM	10 nM
Rhokinase inhibitor Y-27632	AbMole Bioscience, #M1817	10 µM	10 µM

Supplementary Table 2: NK cell donor KIR and HLA typing, and licensing status

NK donor	HLA typing	KIR typing	Licensing status
Donor 1	Bw4+, C1+, C2+	3DL1+, 2DL2+, 2DL3+, 2DL1+	Bw4 – yes, C1 – yes, C2 - yes
Donor 2	Bw4+, C1-, C2+	3DL1+, 2DL2+, 2DL3+, 2DL1+	Bw4 – yes, C1 – no, C2 - yes
Donor 3	Bw4-, C1+, C2+	3DL1+, 2DL2-, 2DL3+, 2DL1+	Bw4 – no, C1 – yes, C2 - yes

Supplementary Table 3: Matched and mismatched conditions between the NK cell donors and organoid cultures

		NK donor 1			*NK donor 2*			*NK donor 3*		
**Organoid**	**HLA typing**	**Bw4 ~ 3DL1**	**C1 ~ 2DL2/3**	**C2 ~ 2DL1**	**Bw4 ~ 3DL1**	**C1 ~ 2DL2/3**	**C2 ~ 2DL1**	**Bw4 ~ 3DL1**	**C1 ~ 2DL2/3**	**C2 ~ 2DL1**
PANCO11a	Bw4-, C1+, C2+	MM	M	M	MM	Unl.	M	Unl.	M	M
PANCO22a	Bw4+, C1+, C2+	M	M	M	M	Unl.	M	Unl.	M	M
PANCO45a	Bw4+, C1-, C2+	M	MM	M	M	Unl.	M	Unl.	MM	M
PANCO51	Bw4-, C1+, C2-	MM	M	MM	MM	Unl.	MM	Unl.	M	MM

M = Matched, MM = Mismatched, Unl.= Unlicensed
